# How ChatGPT writes scientific titles in medical research: structural and content differences compared to human authors

**DOI:** 10.5195/jmla.2026.2266

**Published:** 2026-07-01

**Authors:** Paul Sebo, Bing Nie, Ting Wang

**Affiliations:** 1 paul.seboe@unige.ch, Researcher and Family Physician, University Institute for Primary Care (IuMFE), Faculty of Medicine, University of Geneva, Geneva, Switzerland; 2 icynb@foxmail.com, Researcher, Zhejiang Tongji Vocational College of Science and Technology, Hangzhou, Zhejiang, China; 3 twang2@g.emporia.edu, Researcher, School of Library and Information Management, Emporia State University, Emporia, KS, USA

**Keywords:** Article Title, Artificial Intelligence, AI, ChatGPT, Language Model, Medical Publishing, Scientific Writing

## Abstract

**Objective:**

Scientific article titles play a central role in shaping research visibility, interpretation, and discoverability. With the rise of large language models like ChatGPT, there is growing interest in using AI tools to support title generation, yet little is known about how AI-generated titles differ from those written by human authors. This study compared titles written by original authors to those generated by ChatGPT-4.0 from the same abstracts, focusing on structural and content-level differences.

**Methods:**

Fifty research articles published in 2000, before the advent of generative AI, were randomly selected from ten high-impact general internal medicine journals. For each, the structured abstract was submitted to ChatGPT-4.0 using a standardized prompt to generate a title. Human-written and AI-generated titles were then compared using quantitative measures (word and character counts, punctuation marks) and descriptive content analysis (study design, population descriptors, outcome emphasis, public health or clinical framing, temporal context). Paired statistical tests were applied to assess differences.

**Results:**

ChatGPT-generated titles were significantly longer than human-written titles (median 16 vs. 12.5 words, p-value<0.001) and included more characters and punctuation marks (colons in 100% vs. 30%; p-value<0.001). AI titles more often specified or clarified study design, detailed populations, emphasized outcomes, framed findings in public health or clinical terms, and incorporated temporal context.

**Conclusion:**

ChatGPT-4.0 produces more explicit and structured titles than human authors, emphasizing methodological clarity and content completeness. These findings raise important questions about norms in scientific communication and the need for further research and ethical guidance on AI-assisted writing.

## INTRODUCTION

The title of a scientific article plays a critical role in shaping how research is discovered, interpreted, and disseminated. It is often the first, and sometimes the only, element of a paper that is read, and its structure may affect visibility in indexing databases, search engines, and citation rates [1–8]. An effective title must balance clarity, informativeness, and conciseness [4, 5, 9]. Yet despite its importance, there is no definitive consensus on what constitutes a “good” scientific title, nor on which elements, such as study design, population, outcomes, or rhetorical framing, should be prioritized. Preferences vary by discipline, audience, journal policy, and cultural norms, leaving considerable ambiguity around best practices in title construction [4, 5, 9–12]. In addition, journal editors and reviewers often provide inconsistent guidance [13], and empirical evidence on title effectiveness remains limited.

For health sciences librarians and information professionals, titles are central to how research is found and used. They affect how articles are retrieved in bibliographic databases, discovered in institutional repositories, and interpreted during literature searching. In academic and health sciences settings, librarians help users navigate these variations by teaching how article title wording can influence database retrieval and relevance ranking and by helping refine search strategies when results are overly broad, narrow, or inconsistent. As AI-assisted writing tools become increasingly integrated into scholarly workflows, systematic differences in how titles are constructed may influence indexing patterns, search performance, and research visibility. Understanding whether AI-generated titles differ structurally or linguistically from human-written titles is therefore relevant to librarians’ roles in supporting effective evidence retrieval and responsible AI use in scholarly communication.

Against this backdrop, the emergence of large language models (LLMs), including OpenAI’s ChatGPT, has introduced new tools that can support scientific writing tasks [14–17]. LLMs are trained on vast corpora of texts and can generate fluent, coherent, and contextually appropriate language. They have been studied in several domains of academic writing, including summarizing articles or drafting abstracts [14,18–21], and generating reviewer reports [22,23]. Existing studies show that LLMs can produce coherent scientific text, although concerns remain regarding factual reliability, methodological precision, and the risk of undetected errors or hallucinations [24–26]. Only a small number of studies have examined LLM-generated scientific titles [27,28]. Chen and Eger [27] explored abstract-to-title generation using fine-tuned transformer models on large-scale natural language processing and machine learning datasets, reporting that their best end-to-end systems performed comparably to human-authored titles in human evaluation, although humor-oriented title generation remained substantially more challenging. Rehman et al. [28] fine-tuned multiple pre-trained language models for title generation and found that PEGASUS-large outperformed both general-purpose LLMs and other fine-tuned models across automatic evaluation metrics, while zero-shot GPT-3.5-turbo produced generally accurate and creative titles but did not consistently surpass task-specific fine-tuned models. Together, these studies suggest that transformer-based models can generate fluent and contextually appropriate titles from abstracts, and that fine-tuned models may outperform general-purpose LLMs on automated evaluation metrics; however, performance varies depending on domain, evaluation criteria, and model specialization.

Titles are a distinct genre of scientific writing: unlike manuscripts or abstracts, they are short, highly compressed forms of communication that function both as human-readable summaries and as elements that influence search and retrieval. As previously noted, their wording may affect keyword matching, indexing processes, and how effectively articles surface in bibliographic systems. Any systematic change in title construction, whether produced by humans or LLMs, may therefore affect how easily relevant studies are located or interpreted.

Given the growing interest in using LLMs for scientific writing, it is important to understand whether these models follow the same stylistic conventions as human authors or introduce systematic changes that may influence retrieval and discoverability. The existing literature on AI-generated manuscripts does not address this question, and results from longer free-text writing do not necessarily generalize to short, deliberately structured forms like titles.

To address this gap, the present study compares human-written titles of published scientific articles with titles generated by ChatGPT-4.0 from the same abstracts. Our aim is to identify systematic differences in structural, linguistic, and content-level features, without making normative judgments about which type of title is preferable.

Because titles influence how articles are located in bibliographic systems, these differences have practical implications for health sciences librarians who support evidence retrieval and research visibility. Understanding LLM titling patterns may help inform consultation practices, instructional strategies, and broader discussions about the responsible integration of AI-assisted writing in scientific communication.

## METHODS

### Study context and objectives

We conducted a paired comparative study evaluating structural, linguistic, and content features of titles written by original authors versus titles generated by ChatGPT-4.0 from the same abstracts. The objective was to identify systematic differences in format, style, and content emphasis between the two title types.

### Dataset and article selection

To construct the dataset, we first identified the ten general internal medicine journals with the highest impact factors (IF) in the 2023 Journal Citation Reports (JCR). We focused on general internal medicine because it encompasses a broad range of clinical research topics and article types, making it representative of general medical publishing practices. Selecting ten journals provided sufficient diversity in editorial styles and article content while keeping the sample size manageable for detailed analysis. To ensure consistency and relevance across journals, only those fulfilling all of the following criteria were eligible for inclusion: (1) they publish original research articles and/or systematic reviews; (2) they use structured abstracts for both types of articles; and (3) they have been in continuous publication since at least January 2000. Based on these criteria, the following journals were selected: *The Lancet* (IF 98.4)*, The New England Journal of Medicine* (IF 96.3)*, The BMJ* (IF 93.7)*, JAMA* (IF 63.5), *Archives of Internal Medicine* (IF 22.3)*, Annals of Internal Medicine* (IF 19.6)*, CMAJ* (IF 12.9)*, Journal of Travel Medicine* (IF 9.1)*, Journal of Internal Medicine* (IF 9.0)*, and Mayo Clinic Proceedings* (IF 6.9).

From each journal, one researcher (PS) manually screened the table of contents and article metadata to identify eligible publications, defined as original research articles (reporting primary data and results) or systematic reviews published in the year 2000 with a structured abstract. Research letters, brief reports, and equivalent short-form formats were excluded. After identifying all eligible articles, we used computer-generated randomization to select five per journal, for a final sample of 50 articles. Because content analysis methodology does not prescribe fixed sample sizes for studies of short textual units such as titles [29,30], we selected 50 articles to balance diversity across journals with the feasibility of detailed manual analysis of title structure and content. The year 2000 was chosen to ensure that all original titles were written before the emergence of generative AI-assisted writing tools, eliminating any risk of contamination by machine-generated content. Random selection within each journal was conducted using a computer-generated random number algorithm applied to the list of all eligible publications. To avoid an unintended concentration of articles in a single topic area or study design, one researcher (PS) conducted an informal screening of abstracts and article metadata to verify that the selected articles covered a range of study types and subject areas. This screening was not a formal assessment of representativeness but rather a practical check to ensure basic diversity within the randomly drawn sample. Indeed, we did not further restrict inclusion by medical specialty or subject area, as our goal was to capture the broad range of research published across high-impact general medical journals rather than focus on specific clinical domains that might differ systematically in title conventions or terminology.

### ChatGPT title generation protocol

For each selected article, the abstract was extracted and anonymized. All identifying metadata, including the original title, authors, affiliations, and journal, were removed. Each anonymized abstract was submitted in a new session to ChatGPT-4.0 using the same standardized prompt: “*Write a title for this scientific article based on the abstract below*”. Immediately after entering the prompt, we pasted the full abstract of the selected article.

Each submission was independent (no chat memory), and the outputs were not altered, except for standardizing capitalization: words were converted to lowercase when uppercase was not required (e.g., unless referring to names, countries, or other proper nouns). This setup aimed to reflect a realistic use case where a researcher inputs an abstract and retrieves a title without prompt engineering. The resulting human-written and ChatGPT-generated titles are presented in Table 1.

**Table 1:** Comparison of original and ChatGPT-generated titles for 50 abstracts from scientific articles published in 10 high-impact medical journals

Abstract number	Original title	ChatGPT-generated title	Main difference(s) in ChatGPT title
1	Incidence of diabetes mellitus and impaired glucose tolerance in children and adolescents receiving growth-hormone treatment	Growth hormone treatment in children and risk of diabetes: increased incidence of type 2 but not type 1 diabetes mellitus	Added diabetes type detail and outcome emphasis
2	Population requirement for adult critical-care beds: a prospective quantitative and qualitative study	Estimating population needs for adult critical care: a prospective study of bed requirements in a defined UK region	Added geographic context
3	Variation and trends in incidence of childhood diabetes in Europe	Rising incidence of childhood-onset type 1 diabetes in Europe: findings from the EURODIAB register, 1989–94	Specified disease subtype, time frame, and registry
4	Influence of social network on occurrence of dementia: a community-based longitudinal study	Extent and quality of social networks as predictors of dementia: a community-based cohort study in Sweden	Expanded on network quality and geographic context
5	Effect of centre on outcome of bone-marrow transplantation for acute myeloid leukaemia	Outcomes of allogeneic bone-marrow transplantation for acute myeloid leukaemia: variability between European centres	Added outcome focus and regional comparison
6	Metronidazole to prevent preterm delivery in pregnant women with asymptomatic bacterial vaginosis	Treatment of asymptomatic bacterial vaginosis in pregnancy and risk of preterm delivery: a randomized controlled trial	Added study design
7	A randomized trial of itraconazole in allergic bronchopulmonary aspergillosis	Itraconazole as adjunctive therapy in corticosteroid-dependent allergic bronchopulmonary aspergillosis: a randomized controlled trial	Added therapeutic context and study design
8	Meta-analyses of the relation between silicone breast implants and the risk of connective-tissue diseases	Breast implants and risk of connective-tissue diseases: a meta-analysis of cohort, case-control, and cross-sectional studies	Specified study types in meta-analysis
9	Long-term treatment with a platelet glycoprotein-receptor antagonist after percutaneous coronary revascularization	Oral xemilofiban for long-term prevention of ischemic events after coronary revascularization: a randomized controlled trial	Named specific drug and added study design
10	Cardiopulmonary resuscitation by chest compression alone or with mouth-to-mouth ventilation	Chest compression alone versus with mouth-to-mouth ventilation in bystander CPR: a randomized trial of dispatcher-assisted instructions	Added cardiopulmonary resuscitation (CPR) context and study type
11	Smoking and dementia in male British doctors: prospective study	Smoking and risk of dementia and Alzheimer's disease: findings from a prospective study of British male doctors	Specified Alzheimer’s disease
12	Establishing a standard definition for child overweight and obesity worldwide: international survey	Body mass index cut off points for overweight and obesity in children: international survey	Focused on body mass index (BMI) cutoffs
13	Single blind, randomized trial of efficacy and acceptability of oral picolax versus self-administered phosphate enema in bowel preparation for flexible sigmoidoscopy screening	Bowel preparation for flexible sigmoidoscopy: randomized trial of enema versus Picolax	Simplified procedural comparison and study type
14	Disability in young people and adults one year after head injury: prospective cohort study	Disability after head injury in young people and adults: incidence and outcomes from a one year follow up study	Added outcome follow-up
15	US women's attitudes to false positive mammography results and detection of ductal carcinoma in situ: cross sectional survey	Women’s attitudes toward false positives and detection of ductal carcinoma in situ after screening mammography: a cross sectional survey	Added mammography context
16	Systematic implementation of an advance directive program in nursing homes: a randomized controlled trial	Effect of systematic implementation of advance directives in nursing homes: a randomized controlled trial	N/A
17	Association of sleep-disordered breathing, sleep apnea, and hypertension in a large community-based study	Sleep-disordered breathing and hypertension: findings from the Sleep Heart Health Study	Named cohort
18	Characteristics of child passenger deaths and injuries involving drinking drivers	Child passenger deaths involving drinking drivers: an analysis of risks and prevention opportunities in the United States	Framed prevention opportunity and specified region
19	Physical activity and risk of stroke in women	Physical activity and risk of stroke in women: a prospective study in the Nurses' Health Study cohort	Named cohort and added study design
20	Trends in twin birth outcomes and prenatal care utilization in the United States, 1981-1997	Prenatal care and outcomes in twin pregnancies: trends in preterm birth and infant mortality in the United States, 1981–1997	Emphasized outcomes in context
21	Ten-year trends in hospital care for congestive heart failure: improved outcomes and increased use of resources	Ten-year trends in hospital outcomes for congestive heart failure: declining mortality and length of stay with increased intervention use	Added outcome trends
22	Cigarette smoking and risk of clinically overt thyroid disease: a population-based twin case-control study	Smoking and risk of clinically overt thyroid disease: a population-based twin study	Simplified phrasing
23	Consumption of NSAIDs and the development of congestive heart failure in elderly patients: an underrecognized public health problem	Nonsteroidal anti-inflammatory drugs and risk of first hospital admission for congestive heart failure: a case-control study	Added case-control design and outcome detail
24	Indications for hospitalization of patients with hyperkalemia	Hospitalization for hyperkalemia: current practices and proposed criteria for admission	Clarified clinical context and criteria
25	Screening for hepatitis C virus in a health maintenance organization	Using a database risk algorithm to identify asymptomatic hepatitis C infection: a screening study in a managed care population	Added screening algorithm detail and population
26	Prevalence of and risk factors for hepatic steatosis in Northern Italy	Prevalence and risk factors of hepatic steatosis: stronger association with obesity than with alcohol consumption	Compared obesity vs. alcohol association
27	Dizziness among older adults: a possible geriatric syndrome	Dizziness in older adults: a multifactorial geriatric syndrome with implications for prevention and management	Framed as multifactorial syndrome with implications
28	Invasive and noninvasive strategies for management of suspected ventilator-associated pneumonia. A randomized trial	Invasive versus clinical strategy for managing suspected ventilator-associated pneumonia: a multicenter randomized trial	Framed as invasive vs. clinical strategy
29	Effect of cyclooxygenase-2 inhibition on renal function in elderly persons receiving a low-salt diet. A randomized, controlled trial	Effect of the COX-2 inhibitor rofecoxib on renal function in elderly patients: comparison with nonselective NSAIDs	Named specific cyclo-oxygenase-2 (COX-2) drug, added comparison
30	Reduction in obesity and related comorbid conditions after diet-induced weight loss or exercise-induced weight loss in men. A randomized, controlled trial	Effects of diet- and exercise-induced weight loss on fat distribution and insulin sensitivity in obese men: a randomized controlled trial	Specified outcomes and insulin sensitivity
31	Time required for approval of new drugs in Canada, Australia, Sweden, the United Kingdom and the United States in 1996-1998	International comparison of new drug approval times: Canada lags behind the US, UK, and Sweden	Emphasized international comparison, lag time
32	Incidence and outcomes of diabetes mellitus in elderly people: report from the Canadian Study of Health and Aging	Incidence and outcomes of diabetes mellitus in elderly Canadians: findings from the Canadian Study of Health and Aging	Specified cohort population
33	Otolaryngologists' perceptions of the indications for tympanostomy tube insertion in children	Indications for tympanostomy tube insertion in children: survey of otolaryngologists' practices and agreement in Ontario	Survey focus and regional practice agreement
34	Illness outbreak associated with Escherichia coli O157:H7 in Genoa salami	Escherichia coli O157:H7 outbreak linked to dry fermented salami in Ontario: a case-control and environmental investigation	Added outbreak investigation and location
35	How valid are utilization review tools in assessing appropriate use of acute care beds?	Validity of utilization review tools for assessing appropriateness of hospital care: comparison with expert clinical judgement in cardiology	Added clinical judgment
36	Resources used by general practitioners for advising travelers from New Zealand	Resources used by general practitioners for travel health advice in New Zealand: a survey of usefulness and utilization	Survey utility
37	Reported side effects to chloroquine, chloroquine plus proguanil, and mefloquine as chemoprophylaxis against malaria in Danish travelers	Self-reported symptoms and compliance with malaria prophylaxis: comparison of chloroquine, chloroquine plus proguanil, and mefloquine in Danish travelers	Added compliance and comparative symptoms
38	Cabin location and the likelihood of motion sickness in cruise ship passengers	Cabin location and risk of motion sickness at sea: no association found in passengers during rough conditions	Framed no association in context of conditions
39	How important a priority is travel medicine for a typical British family practice?	Illness and compliance among long-haul travelers: a retrospective study from a UK primary care travel clinic	Framed illness/compliance, added retrospective detail
40	Risks of hepatitis B in travelers as compared to immunization status	Hepatitis B risk and vaccination status among European travelers: a cross-sectional survey across nine countries	Specified regions, added vaccination focus and study design
41	Independent effects of obesity and cortisol in predicting cardiovascular risk factors in men and women	Sex-specific associations of plasma cortisol and obesity with cardiovascular risk: results from the MONICA study	Sex-specific outcome associations emphasized
42	Dietary and other non-pharmacological treatments in patients with drug-treated hypertension and control subjects	Nutrient intake and lifestyle patterns in drug-treated hypertensives: a population-based study from Finland	Specified lifestyle patterns and study base
43	Mechanisms behind gender differences in circulating leptin levels	Gender differences in leptin levels and adipose tissue leptin secretion: independent effects beyond body fat content	Specified leptin physiology and independence
44	Mortality, risk indicators of death, mode of death and symptoms of angina pectoris during 5 years after coronary artery bypass grafting in men and women	Sex differences in five-year outcomes after coronary artery bypass grafting: mortality, risk factors, and angina symptoms in a population-based cohort	Framed coronary artery bypass grafting (CABG) outcomes by sex and time, added study design
45	Myocardial hypertrophy in transgenic mice overexpressing the bovine growth hormone (bGH) gene	Growth hormone–induced myocardial hypertrophy in transgenic mice: structural adaptations and downregulation of muscarinic receptors	Added receptor findings and myocardial detail
46	Poststreptococcal reactive arthritis in adults: a case series	Poststreptococcal reactive arthritis in adults: clinical features, diagnosis, and treatment response in a retrospective cohort	Added clinical course and retrospective label
47	Detectable blood alcohol after a motor vehicle crash and screening for alcohol abuse/dependence	Alcohol abuse screening after motor vehicle crashes: missed opportunities for intervention in hospitalized patients with detectable blood alcohol levels	Emphasized missed interventions post-crash
48	Outcomes of primary and secondary treatment of pericardial effusion in patients with malignancy	Management of malignancy-related pericardial effusion: outcomes of pericardiocentesis with extended drainage and surgical approaches	Added management detail and procedural scope
49	Differentiation of typhoid fever from fulminant hepatic failure in patients presenting with jaundice and encephalopathy	Early diagnosis of typhoid fever in patients with jaundice and encephalopathy: clinical and laboratory predictors and treatment outcomes	Added predictors and outcomes in encephalopathy
50	Renal cell carcinoma metastatic to the pancreas: clinical and radiological features	Pancreatic metastases from renal cell carcinoma: clinical features, imaging characteristics, and treatment outcomes	Added treatment outcomes

### Structural, linguistic, and content-level comparison

For each abstract, the original title and the ChatGPT-generated title were compared on a set of predefined structural features: total number of words, total number of characters, and punctuation marks (colons, semicolons, question marks). These features were selected because they represent widely studied and quantifiable structural elements linked to variation in scientific title and impact, making them an appropriate initial basis for comparing human- and AI-generated title construction. Specifically, while longer titles are not inherently more visible in search systems, including more specific search terms can improve indexing and keyword matching in academic databases [31]. Similarly, punctuation in academic titles has le potential to attract specific groups of readers, but evidence regarding its impact on retrieval and citation is mixed [4,32].

In parallel, a descriptive coding framework was developed inductively to capture recurrent linguistic and content-level differences between the two types of titles. This framework was informed by prior studies describing common features of scientific titles, such as the inclusion of study design, outcomes, population descriptors, and framing elements [33–36]. However, as noted in the Introduction, there is no consensus on what constitutes a ‘good’ title or which elements should be prioritized. Against this background, we focused on the following thematic elements, which are frequently discussed in the literature and observed in practice: addition or clarification of study design, outcome emphasis, specification of the study population (population descriptors), terminology simplification, inclusion of specific intervention or diagnostic terms, public health or clinical framing, and temporal framing [34–37]. Each adjustment identified in the ChatGPT-generated title was cross-checked against the corresponding abstract to verify that it accurately reflected the study’s content. Each title was independently coded and verified by two reviewers (PS and TW) to ensure consistency and accuracy. Disagreements were resolved by discussion and consensus. The full text of the articles was not consulted for this study; all assessments were limited to information available in the abstract, as this was the source on which ChatGPT generated the titles.

### Statistical analysis

All extracted data were first entered into Microsoft Excel (Microsoft Corporation, Redmond, WA, USA) and then exported to Stata for analysis. Structural variables were defined as total word count, total character count, and the presence of specific punctuation marks (colons, semicolons, question marks). Linguistic and content-level variables were defined according to the thematic framework described above (e.g., mention or clarification of study design, outcome emphasis, population descriptors, terminology simplification, intervention or diagnostic specification, public health or clinical framing, and temporal framing).

Descriptive statistics were reported as medians with interquartile ranges (IQRs) for continuous variables (word count, character count) and as frequencies with percentages for categorical variables (presence of colon, semicolon, or question format). Paired comparisons between human-written and ChatGPT-generated titles were conducted. The Wilcoxon signed-rank test was applied to continuous variables, while McNemar’s test was applied to categorical variables. Linguistic and content-level variables were summarized descriptively. All analyses were conducted using Stata version 15.1 (StataCorp, College Station, TX, USA). A two-sided p-value <0.05 was considered statistically significant.

### Ethical considerations

This study used only publicly available research articles and did not involve human participants, patient data, or confidential information; therefore, ethics committee approval was not required. The use of ChatGPT complied with OpenAI’s terms of service.

## RESULTS

The main results of the study are summarized in four tables. Tables 1 and 2 support the descriptive thematic analysis: Table 1 presents the original and ChatGPT-generated titles for each abstract, along with brief summaries of their key differences; Table 2 synthesizes the main thematic patterns observed across abstracts, corresponding to recurrent linguistic, content-level, and framing differences. Tables 3 and 4 support the quantitative analysis: Table 3 displays the raw structural data extracted from each pair of titles, while Table 4 reports descriptive statistics and p-values comparing human- and AI-generated titles.

**Table 2:** Main systematic differences observed in ChatGPT-generated titles compared to original titles for 50 abstracts from scientific articles published in 10 high-impact medical journals.

Main Differences Observed in ChatGPT Titles	Description	Examples	Abstract numbers	Frequency, n (%)
Addition of methodology or study design	ChatGPT frequently added or clarified the type of study	e.g., “a randomized controlled trial”, “a case-control study”	6, 7, 8, 9, 10, 19, 23, 29, 33, 34, 35, 36, 39, 40, 42, 44, 46	17 (34)
Specification or emphasis on outcomes (outcome emphasis)	Titles were reworded to highlight study outcomes, conclusions, or provide more detail	e.g., “increased incidence”, “body mass index cut off points”	1, 3, 5, 12, 14, 20, 21, 23, 26, 30, 31, 37, 38, 39, 40, 41, 43, 45, 46, 49, 50	21 (42)
Clarification or expansion of population descriptors	ChatGPT often added details about age, sex, geography, or cohort name	e.g., “a defined UK region”, “EURODIAB register”	2, 3, 4, 5, 10, 17, 18, 19, 25, 32, 33, 34, 39, 40, 41, 42, 47	17 (34)
Terminology simplification	Some titles were rephrased for clarity, fluency, and conciseness	e.g., “Bowel preparation for flexible sigmoidoscopy: randomized trial of enema versus Picolax”	13, 22	2 (4)
Addition or clarification of interventions or diagnostic terms	ChatGPT explicitly named specific treatments, drugs, or physiological details, or added precision about the intervention	e.g., “itraconazole as adjunctive therapy”, “xemilofiban”, “muscarinic receptors”	7, 9, 28, 29, 45, 48	6 (12)
Public health or clinical framing	Some titles introduced language emphasizing clinical or public health implications	e.g., “implications for prevention”, “missed opportunities for intervention”	18, 27, 47	3 (6)
Temporal framing	Where relevant, ChatGPT added or clarified the temporal context	e.g., “1989-94”, “one year follow up study”	3, 14, 44	3 (6)

**Table 3:** Comparison of title length and punctuation features in original and ChatGPT-generated titles for 50 abstracts from scientific articles published in 10 high-impact medical journals

Abstract number	Original title - number of words	ChatGPT title - number of words	Original title - number of characters	ChatGPT title - number of characters	Original title - contains a colon (Y/N)	ChatGPT title - contains a colon (Y/N)	Original title - contains a semicolon (Y/N)	ChatGPT title - contains a semicolon (Y/N)	Original title - is a question (Y/N)	ChatGPT title - is a question (Y/N)
1	15	20	124	121	N	Y	N	N	N	N
2	12	18	101	115	Y	Y	N	N	N	N
3	10	15	65	107	N	Y	N	N	N	N
4	12	16	91	105	Y	Y	N	N	N	N
5	12	13	86	116	N	Y	N	N	N	N
6	12	16	97	118	N	Y	N	N	N	N
7	9	13	77	133	N	Y	N	N	N	N
8	14	15	105	124	N	Y	N	N	N	N
9	11	15	114	124	N	Y	N	N	N	N
10	10	16	91	135	N	Y	N	N	N	N
11	9	17	63	111	Y	Y	N	N	N	N
12	12	14	99	91	Y	Y	N	N	N	N
13	23	11	176	86	N	Y	N	N	N	N
14	14	19	90	111	Y	Y	N	N	N	N
15	18	19	124	136	Y	Y	N	N	N	N
16	14	14	105	105	Y	Y	N	N	N	N
17	13	11	105	87	N	Y	N	N	N	N
18	10	17	81	121	N	Y	N	N	N	N
19	8	17	45	101	N	Y	N	N	N	N
20	14	19	91	124	N	Y	N	N	N	N
21	16	19	111	137	Y	Y	N	N	N	N
22	14	12	106	83	Y	Y	N	N	N	N
23	18	16	131	124	Y	Y	N	N	N	N
24	7	10	61	87	N	Y	N	N	N	N
25	10	19	68	126	N	Y	N	N	N	N
26	11	15	70	113	N	Y	N	N	N	N
27	8	14	59	110	Y	Y	N	N	N	N
28	13	13	115	120	N	Y	N	N	N	N
29	18	16	132	114	N	Y	N	N	N	N
30	21	20	154	137	N	Y	N	N	N	N
31	20	15	123	94	N	Y	N	N	N	N
32	18	18	113	118	Y	Y	N	N	N	N
33	11	15	93	120	N	Y	N	N	N	N
34	10	16	73	123	Y	Y	N	N	N	N
35	14	18	87	140	N	Y	N	N	Y	N
36	11	18	79	119	N	Y	N	N	N	N
37	17	18	136	153	N	Y	N	N	N	N
38	12	17	78	109	N	Y	N	N	N	N
39	13	16	82	108	N	Y	N	N	Y	N
40	11	15	68	112	N	Y	N	N	N	N
41	15	15	102	112	N	Y	N	N	N	N
42	13	13	112	107	N	Y	N	N	N	N
43	8	16	65	116	N	Y	N	N	N	N
44	25	20	152	150	N	Y	N	N	N	N
45	12	14	93	131	N	Y	N	N	N	N
46	8	15	61	126	Y	Y	N	N	N	N
47	13	19	95	152	N	Y	N	N	N	N
48	13	14	95	132	N	Y	N	N	N	N
49	15	18	119	136	N	Y	N	N	N	N
50	11	13	83	115	Y	Y	N	N	N	N

**Table 4:** Summary of differences in title length and punctuation features between original and ChatGPT-generated titles for 50 abstracts from scientific articles published in 10 high-impact medical journals

	Total number of words	Median number of words (interquartile range, min-max)	Total number of characters	Median number of characters (interquartile range, min-max)	Number of titles with a colon (%)	Number of titles with a semicolon (%)	Number of titles that are questions (%)
Original titles (n=50)	658	12.5 (11-15, 7-25)	4846	94 (78-113, 45-176)	15 (30)	0 (0)	2 (4)
ChatGPT titles (n=50)	792	16 (14-18, 10-20)	5895	118 (109-126, 83-153)	50 (100)	0 (0)	0 (0)
p-value		<0.001^1^		<0.001^1^	<0.001^2^	N/A	0.50^3^

1 Wilcoxon signed-rank test

2 McNemar’s test

3 Exact McNemar’s test

### Quantitative comparison of title structure

ChatGPT-generated titles were generally longer than those created by humans. The median word count for AI-generated titles was 16 (IQR: 14–18), compared to 12.5 (IQR: 11–15) for human-written titles (p-value <0.001). Similarly, the median number of characters was higher for ChatGPT titles (118; IQR: 109–126) than for human-written ones (94; IQR: 78–113; p-value <0.001). All ChatGPT-generated titles included a colon, compared to only 30% of human-written titles (p-value <0.001). Only two human-written titles were phrased as questions, compared to none among ChatGPT titles, which was not statistically significant.

### Descriptive comparison of content and framing

Systematic differences were identified in the way ChatGPT-4.0 generated titles from abstracts, as summarized in Table 2. A prominent pattern was the frequent addition or clarification of methodological information, particularly through more explicit identification of study design (17 titles, 34%). Titles produced by ChatGPT also tended to emphasize outcomes or key findings more explicitly than their human-written counterparts (21 titles, 42%). For example, these titles included terms such as “increased incidence” (abstract 1) or detailed findings like “body mass index cut off points” (abstract 12). Another recurring modification was the clarification or expansion of the study population, with ChatGPT specifying age groups, geographic settings, or cohort names (e.g., “a defined UK region” in abstract 2 or “EURODIAB register” in abstract 3) (17 titles, 34%). Some AI-generated titles underwent terminology simplification, replacing technical or compound phrasing with simpler and more common wording (e.g., abstract 13: “single blind, randomized trial of efficacy and acceptability of oral picolax versus self-administered phosphate enema in bowel preparation for flexible sigmoidoscopy screening” simplified to “bowel preparation for flexible sigmoidoscopy: randomized trial of enema versus Picolax”) (2 titles, 4%). This pattern reflects ChatGPT’s general tendency to favor fluency and accessibility, consistent with prior observations that LLMs often prioritize readability over maintaining domain-specific precision [38–40].

Other AI-generated titles included the addition or clarification of specific interventions, drugs, or diagnostic elements, such as naming a drug like “xemilofiban” (abstract 9) or specifying “muscarinic receptors” (abstract 45) (6 titles, 12%). In several cases, the AI titles framed findings in terms of public health or clinical framing, introducing expressions like “implications for prevention” (abstract 27) or “missed opportunities for intervention” (abstract 47) (3 titles, 6%). Finally, the temporal framing was occasionally added or clarified, as seen in examples such as “1989–94” (abstract 3) or “one year follow up study” (abstract 14) (3 titles, 6%).

Together, these patterns illustrate ChatGPT’s tendency to produce more structured, explicit, and information-dense titles. All thematic additions and modifications introduced by ChatGPT accurately reflected the information contained in the corresponding abstracts. No instances of factual inaccuracy or misrepresentation were identified during reviewer verification.

## DISCUSSION

### Summary of key findings

This study revealed systematic differences between titles written by human authors and those generated by ChatGPT-4.0 from the same scientific abstracts. Across a matched dataset of 50 original medical research articles, ChatGPT-generated titles were consistently longer, more structured, and more explicit in content than their human-written counterparts.

Quantitatively, AI-generated titles included significantly more words and characters and systematically used colons to structure information. Descriptively, ChatGPT displayed consistent tendencies to specify or clarify the study design, emphasize key outcomes, clarify or expand population descriptors, frame results in terms of public health or clinical framing, and incorporate temporal context. Terminological simplifications and the addition of contextual details were also frequently observed. These two tendencies, adding contextual specificity while simplifying technical phrasing, suggest that ChatGPT favors titles that are simultaneously more explicit and more accessible.

However, the greater explicitness and structural regularity observed in ChatGPT titles came with trade-offs. Human-written titles often used concise phrasing, disciplinary shorthand, or more narrative or question-based structures that conveyed tone, emphasis, or conceptual stance, elements largely absent from AI-generated versions. Several ChatGPT titles also introduced more detail than may be appropriate under journal title-length constraints, and the consistent use of a two-part colon structure produced titles that were more formulaic and uniform. Thus, while AI-generated titles were more standardized and explicit, they may be less aligned with journal-specific style expectations and disciplinary conventions regarding conciseness, framing, and authorial voice.

### Comparison with the literature

To date, a growing number of empirical studies have examined how LLMs perform in the task of scientific text generation [14–23,27,28]. Most published work has focused on broader applications of LLMs in academic writing, including abstract generation [14,18–21], literature review synthesis [41,42], and peer review drafting [22,23]. Within the more specific domain of title generation, very few studies have directly addressed this task [27,28]. To our knowledge, our work is the first to conduct a paired, descriptive, and quantitative comparison between human-written titles and ChatGPT-generated titles derived from the same scientific abstracts. By combining structural features with thematic analysis, this study offers a novel empirical contribution to understanding how LLMs address a critical yet underexplored component of scientific communication.

Chen and Eger (2023) evaluated the ability of transformer-based models, including ChatGPT, to generate scientific titles from abstracts in the fields of natural language processing and machine learning [27]. Their study emphasized stylistic dimensions, such as humor and novelty, and introduced the first large-scale dataset of humorous scientific titles. While some models (e.g., BARTxsum) approached human-level quality, capturing genuine humor remained a significant challenge. This contrasts with our findings: we did not evaluate creativity or humor but instead observed that ChatGPT produced more explicit, structured, and information-dense titles, often prioritizing methodological transparency over stylistic variation.

Rehman et al. (2024) assessed the capacity of LLMs to generate titles from abstracts, comparing the performance of fine-tuned models with large general-purpose models in zero-shot settings [28]. They report that a fine-tuned PEGASUS-large model outperformed GPT-3.5-turbo and LLaMA-3-8B on standard automated metrics, and human annotators often favored its outputs. While they noted that ChatGPT could generate creative titles in different stylistic modes, our study extends their findings by providing a paired, article-level comparison with human-written titles, highlighting not only structural features but also systematic differences in framing and emphasis.

### Implications for practice and research

Our findings have implications for health sciences librarians, particularly in settings where they support researchers in developing effective search strategies and understanding how title wording may influence indexing and retrieval in bibliographic databases. Because titles are key elements used in bibliographic indexing and search algorithms, changes in title structure or terminology may directly affect how articles are classified and retrieved. In particular, AI-generated titles may increase retrieval performance in keyword-based systems by explicitly naming study designs, populations, and outcomes. Such information may facilitate controlled vocabulary indexing (e.g., MeSH in PubMed) and improve keyword matching in searchable title fields in bibliographic databases and platforms such as PubMed, Scopus, and Web of Science. Moreover, this approach aligns with the PICO framework commonly used in medical information retrieval [43,44]. However, broader or more inclusive labels may also lead to less precise retrieval. According to Manning et al., precision is defined as the proportion of retrieved documents that are relevant [45]. From an information retrieval perspective, titles containing a larger number of keywords may generate a greater number of indexed terms, which can broaden retrieval and increase the likelihood of spurious matches. In some cases, this may introduce additional noise and potentially reduce precision in search results. Given these possibilities, librarians can help researchers understand how AI-generated titles may influence indexing consistency and retrieval accuracy, offer targeted instruction on how title wording shapes search strategies and retrieval results, and contribute to institutional discussions about policies on AI-assisted writing.

Librarians are also well positioned to advise on disclosure practices and to monitor how AI-generated titles interact with bibliographic systems over time. As indexing increasingly incorporates algorithmic or AI-assisted components, including machine learning and LLM–based approaches to subject assignment and semantic analysis, the interaction between AI-generated titles and AI-driven indexing systems warrants attention. If titles are produced using models that systematically foreground study design, population descriptors, or outcome language, and if indexing systems likewise rely on automated term extraction or semantic pattern recognition, these features may be reinforced during the indexing process. Conversely, shifts in titling conventions may influence how AI-based indexing models interpret and classify content. Understanding this evolving interaction between AI-assisted writing and AI-assisted indexing will be important for maintaining accurate, transparent, and effective information retrieval in bibliographic systems.

The observed differences highlight the need for clearer and more explicit guidance as AI becomes integrated into scholarly writing practices. While many journals may implicitly favor brevity and certain stylistic conventions in titles, such as concise phrasing, rhetorical variation, or avoidance of overly formulaic structures, these expectations are often shaped by disciplinary convention rather than by formal policy. For health sciences librarians who support researchers in understanding publication norms, greater transparency around criteria for title length, punctuation, and descriptive structure would facilitate more consistent and informed guidance, particularly when AI-assisted tools are used in the writing process. Although titles are unlikely to determine acceptance on their own, they are highly visible elements of scholarly communication that influence how research is presented, interpreted, and retrieved. Clearer articulation of expectations surrounding AI-assisted titling would therefore assist librarians in their instructional roles.

Ethical considerations are also central. Current authorship and AI-use guidelines apply broadly to the writing process and rarely address titles explicitly [46–50]. However, because titles function as core bibliographic metadata and are used directly in indexing, retrieval, and search ranking, transparency about AI involvement is particularly relevant at the title level. Librarians increasingly engage in institutional conversations about research integrity, AI literacy, and transparency in scholarly communication. Awareness of how AI-generated elements appear in highly visible metadata fields such as titles may therefore inform local guidance, training initiatives, and policy discussions about responsible AI integration in academic writing.

Our findings highlight several areas where further evidence is needed to support librarians’ instructional and consultative work. While completeness and structure may improve information retrieval, they do not necessarily align with how titles influence impact. Several studies have shown that shorter titles and titles without colons are associated with higher citation rates and visibility [2–5,51], although some findings, such as those reported by Jacques and Sebire [1], as well as by Rostami et al. [52], suggest the opposite. Our findings therefore raise an important and unresolved question: since ChatGPT-generated titles are consistently longer, more structured, and more likely to contain colons, will they enhance visibility through improved indexing and keyword matching, or reduce citation potential due to lower readability and engagement? This paradox between completeness and conciseness underscores the open question of whether AI-generated titles will improve discoverability at the cost of readability or reader engagement. In addition, foregrounding design, outcomes, or populations may predispose readers to particular interpretations, narrowing perceived generalizability or overstating certainty. These framing effects reflect defaults of the model rather than deliberate authorial choices and may shape how research is received and cited. Addressing such risks will require both empirical study and clear editorial guidance.

For researchers, LLMs such as ChatGPT may serve as useful co-writing tools to enhance the completeness, precision, or discoverability of titles, particularly for early-career researchers or non-native English speakers [53]. ChatGPT-generated titles consistently included key structural features such as study design, population descriptors, and outcome emphasis. These features reflect established conventions in scientific titling that aim to convey clarity and specificity, but their inclusion does not necessarily imply greater effectiveness or appropriateness. However, such structural patterns may improve visibility by aligning with keyword-based retrieval in academic databases and general search engines [54]. At the same time, longer or more formulaic titles could introduce framing bias that narrows interpretation, and the tendency to generate longer, more structured titles raises concerns about readability while enhancing searchability [2,13,52]. This paradox between discoverability and accessibility highlights an important intersection between AI-assisted title generation, information retrieval, and search engine optimization (SEO) practices. Against this backdrop, researchers might consider using LLMs as first draft generators while applying their own judgment to refine titles for clarity, tone, and balance.

While ChatGPT occasionally simplified technical or compound terminology, these changes were not always neutral. For example, simplification could result in the omission of methodological or outcome details, thereby reducing informational completeness. This tension between clarity and precision illustrates the need for human oversight in evaluating whether AI-generated adjustments accurately reflect the study’s content. Future research should further explore how such simplifications affect reader understanding, perceived rigor, and information retrieval.

### Limitations

This study has several limitations. First, it was conducted on a historical dataset of articles published in 2000, prior to the emergence of LLMs, to ensure that original titles were entirely human-written. While this provides a clean comparison, some editorial conventions may have evolved over the past two decades, potentially limiting generalizability to contemporary writing. Second, title conventions in our dataset may have been shaped by journal-specific editorial policies, such as restrictions on title length or discouragement of colons. At the time, the ICMJE Uniform Requirements recommended only that titles be ‘concise but informative’, without prescribing detailed structural rules [55,56]. The observed differences between ChatGPT- and human-written titles may therefore partly reflect contrasts between journal house styles and ChatGPT’s default titling patterns, rather than solely fundamental differences in emphasis. Third, we used only structured abstracts as input and operated ChatGPT in a zero-shot mode without prompt optimization or chain-of-thought reasoning. As such, our findings reflect the model's baseline performance rather than its full capabilities when used by experienced prompt engineers. Fourth, while we employed rigorous manual coding, certain content judgments, such as what constitutes an “outcome mention” or a “population descriptor”, may involve interpretive nuance. Fifth, we focused exclusively on high-impact general medical journals; patterns may differ in specialty fields, lower-impact venues, or non-English publications. Sixth, the diversity check performed on the randomly selected articles was informal and not based on a standardized taxonomy of study designs or subject areas. As such, although the sample covered a range of topics, it cannot be considered formally representative of the full spectrum of research published in general medical journals. Seventh, this study did not include reader ratings or perceptions. A follow-up study is planned in which researchers will compare the accuracy and appeal of human-written versus ChatGPT-generated titles based on the same abstracts. Finally, article selection and the representativeness check were performed by a single researcher (PS), which may introduce bias despite the use of randomization.

### Conclusion

This study provides empirical evidence that ChatGPT-4.0, when prompted in a zero-shot setting using only structured abstracts, produces article titles that are systematically longer, more detailed, and more likely to include key elements such as study design, population descriptors, and outcome emphasis compared to human-written titles. These results suggest that LLMs can support scientific writing by enhancing structural completeness and transparency, although human input may still be necessary to refine tone, style, and rhetorical nuance. At the same time, longer and more formulaic titles may reduce stylistic conciseness, and simplification of terminology may omit technical specificity or introduce unnecessary detail, making titles less aligned with journal-specific conventions. These trade-offs reinforce the need for careful human oversight when AI is used in title construction.

As generative AI tools become increasingly integrated into academic workflows, further research is warranted to explore their impact on readers’ perceptions, editorial standards, and the evolving norms of scientific communication. Ethical considerations, including transparency in authorship, acknowledgment of AI assistance, and adherence to journal guidelines, will also need to be addressed as LLM-assisted writing becomes more widespread.

## Data Availability

The data associated with this article are available in Table 1.
